# The Efficacy of Compression Stockings on Patients With Nocturia: A Single-Arm Pilot Study

**DOI:** 10.7759/cureus.28603

**Published:** 2022-08-30

**Authors:** Kanya Kaga, Tomonori Yamanishi, Chiharu Shibata, Tomohiko Kamasako, Mayuko Kaga, Miki Fuse

**Affiliations:** 1 Department of Urology, Chiba Prefectural Sawara Hospital, Katori, JPN; 2 Department of Urology, Continence Center, Dokkyo Medical University Hospital, Mibu, JPN; 3 Department of Urology, Utsunomiya Neurospine Center Symphony Clinic, Utsunomiya, JPN; 4 Department of Urology, Saiseikai Utsunomiya Hospital, Utsunomiya, JPN; 5 Department of Urology, Mihama Narita Clinic, Narita, JPN; 6 Department of Urology, Yokohama Rosai Hospital, Yokohama, JPN

**Keywords:** nocturia, night-time frequency, hours of undisturbed sleep, frequency-volume chart, compression stockings

## Abstract

Objectives: Behavioral treatment for nocturia includes wearing compression stockings. However, a reading of the cited literature for evidence shows that there is not enough research data to support this recommendation, and it is controversial. The present study aimed to investigate and supplement evidence on the effects of wearing compression stockings during the daytime in patients with nocturia.

Methods: This was a single-arm prospective study to investigate the effects of compression stockings on nocturia for four weeks. Patients were asked to record a frequency-volume chart and complete various questionnaires at baseline and after four weeks, and also provide feedback on treatment satisfaction. The primary endpoint was a change in night-time frequency in the frequency-volume chart from the baseline to the end of treatment.

Results: Thirty-four patients (19 men and 15 women; age: 72.3 ± 12.6 years) were included. Two patients dropped out because of pain associated with wearing compression stockings and one due to a refusal to wear compression stockings every day. Therefore 31 patients were analyzed. In the frequency-volume chart, night-time and 24-hour frequencies significantly decreased by 0.5 and 1.1 episodes, respectively (P = 0.004 and P = 0.035, respectively). The hours of undisturbed sleep significantly increased by 0.8 h (P = 0.013). No significant differences were observed in nocturnal or 24-h urine volumes, the number of urgency or urinary incontinence episodes, the mean or maximum voided volume, the nocturnal polyuria index, or the first night-time voided volume. The total overactive bladder symptom score significantly decreased (P = 0.006). Significant reductions were also observed in all overactive bladder symptom score subscores, except for the daytime frequency score.

Conclusion: The present results suggest the effectiveness of wearing compression stockings during the day was satisfactory in most patients with nocturia, and the treatment was safely continued in patients who experienced no pain when wearing the stockings. Based on the results of this study, we believe that it is worth considering as a treatment for nocturia.

## Introduction

Nocturia is a complex condition that is characterized by nocturnal polyuria, decreased bladder storage function, and sleep disturbance. Nocturnal polyuria is caused by several factors, including congestive heart failure, diabetes mellitus, obstructive sleep apnea, peripheral edema, anxiety disorders, and urologic disease [1]. It has been associated with falls, fractures, and mortality [2,3]. Behavioral therapies include lifestyle interventions and scheduled voiding regimens, including bladder training and pelvic floor muscle training. These therapies have been recommended as the first-line conservative treatment for lower urinary tract symptoms because they are non-invasive and cost-effective. Among these behavioral therapies, wearing compression stockings has been proposed as a treatment option, in addition to lifestyle interventions such as limiting night-time drinking and avoiding caffeine and alcohol. Compression stockings have been widely used as a tool in compression therapies for deep vein thrombosis, varix, lymphedema, and orthostatic hypotension [4-7]. They have been recommended for the treatment of nocturia, particularly nocturnal because they attenuate edema and decrease the accumulation of extracellular fluid in the lower extremities [8,9]. The compression stockings may be safe and effective for the treatment of nocturia, especially for nocturnal polyuria, when they wear in the daytime, decreasing the extra fluid accumulation in the extremities. However, a reading of the cited literature for evidence shows that there is not enough research data to support this recommendation, and it is controversial [1,8].

The present study aimed to investigate and supplement evidence on the effects of wearing compression stockings in the daytime on nocturia.

## Materials and methods

A single-arm prospective analysis of the effects of compression stockings on nocturia for four weeks was performed. All procedures were conducted following the Declaration of Helsinki (UMIN000047192) and with the approval of the Institutional Review Board of Dokkyo Medical University (approval number: R-42-15J). Signed informed consent was obtained from all patients before the initiation of treatment.

The present study enrolled patients with nocturia who had not previously used compression stockings. Nocturia was defined as a condition in which the patient wakes up to urinate at least once per night. To match actual practice conditions, patients with lower urinary tract dysfunction were included, but only those whose condition had been stable for at least three months. In addition, drugs that had been used concomitantly before participation in the study were not changed in dose or withdrawn during the study period. Patients with hypersensitivity to the material of compression stockings, urinary tract infection, lower urinary tract stones, urethral disease, atherosclerosis, and peripheral arterial disease were excluded. Three-day frequency-volume chart (FVC), overactive bladder symptom score (OABSS), and international consultation on the incontinence questionnaire-short form (ICIQ-SF) were recorded by each patient at the baseline and after four weeks of treatment. Patient-reported satisfaction levels after four weeks of treatment were as follows: satisfied, somewhat satisfied, neither satisfied nor dissatisfied, somewhat dissatisfied, and dissatisfied.

The primary endpoint was a change in night-time frequency in the FVC from the baseline to the end of treatment. The secondary endpoints were changed in the following parameters from the baseline to the end of treatment: nocturnal and 24-h urine volumes, 24-h frequency, the number of daily urgencies and incontinence episodes, mean and maximum voided volumes, nocturnal polyuria index (NPi), hours of undisturbed sleep (HUS), and first night-time voided volume in a three-day FVC, OABSS, ICIQ-SF, and ankle and calf circumferences were evaluated. A subanalysis was also performed in patients with nocturia with nocturnal polyuria.

Solve Corp. provided the compression stockings used in the present study, which were made of THUASNE VENOFLEX FAST COTON® (Paris, France). The compression pressure of these stockings ranges between 15 and 20 mmHg, which is slightly higher than that of commercial compression stockings. Appropriate sizes were selected based on the ankle and calf circumferences of each patient according to the documentation accompanying the stockings. Patients were instructed to wear compression stockings except for bedtime at night.

Statistical analysis

Results are shown as the mean ± standard deviation (SD). The Wilcoxon matched-pairs signed-rank test was performed to examine the significance of changes in parameters between the baseline and after four weeks of treatment. P-value < 0.05 was considered to indicate a significant difference. Since no previous studies have established a strict number of cases, the number of recruited patient was set at 30, referring to Sone's study [10].

## Results

A total of 34 patients (19 men, and 15 women; aged 72.3 ± 12.6 years) were included in the present study. The background characteristics of patients are shown in Table 1. 

**Table 1:** Baseline characteristics of patients with nocturia who participated in this study

Characteristics	N = 34
	Mean (SD)
Age, years	72.3 (12.6)
Male/female	19/15
Body weight, kg	60.4 (8.7)
Body mass index, kg/m^2^	23.5 (3.4)
Estimated glomerular filtration rate, mL/min/1.73m^2^	64.2 (21.7)
Brain natriuretic peptide, pg/mL	96.8 (117.1)
Free uroflowmetry	-
Voided volume, mL	100.0 (82.7)
Maximum flow rate, mL/s	10.6 (8.3)
Mean flow rate, mL/s	5.8 (4.0)
Post void residual, mL	19.1 (17.8)
Complications	N = 11
Diabetes mellitus	5
Spinal cord disease	4
Cerebrovascular disease	3
Heart disease	3
Sleep apnea syndrome	2
Peripheral nerve disease	2
Postoperative benign prostatic hyperplasia	1
Concomitant medications	N = 15
Alpha blocker	8
Beta 3 agonist	4
Anticholinergic drug	3
Five alpha reductase inhibitor	2
Phosphodiesterase 5 inhibitor	1

Based on the free uroflow results, the amount of post void residual was low and no patients with the urinary frequency associated with dysuria were observed. Patients with various complications were admitted, but all were in stable condition. They had also been taking concomitant medications for more than three months before the study, and all had problems with nocturia.

Two patients dropped out because of pain associated with wearing compression stockings, and one due to a refusal to wear compression stockings every day. No other adverse events were noted. Therefore 31 patients were examined in the present study.

In the FVC, night-time and 24-h frequencies significantly decreased by 0.5 episodes and 1.1 episodes, respectively (P = 0.004 and P = 0.035, respectively). No significant differences were observed in nocturnal or 24-h urine volumes, urgency or urinary incontinence episodes, mean or maximum voided volumes, NPi, or first night-time voided volumes. HUS significantly increased by 0.8 h (P = 0.013). Total OABSS significantly decreased (P = 0.006). Significant reductions were detected in all OABSS subscores, except for the daytime frequency score. Furthermore, the total score for ICIQ-SF did not significantly decrease (Table 2).

**Table 2:** FVC, OABSS, ICIQ-SF, and ankle/calf circumference at baseline and after four weeks FVC: frequency volume chart; OABSS: overactive bladder symptom score; ICIQ-SF: international consultation on incontinence questionnaire-short form

N = 31; mean (SD)	Before	After	P-value
FVC			
Night-time frequency	2.9 (1.1)	2.4 (1.2)	0.004
Twenty-four-hour frequency	13.1 (2.9)	12.0 (3.5)	0.035
Number of urgency episodes/24 h, N = 8	0.9 (2.0)	0.5 (1.2)	0.312
Number of leaks/24 h, N = 7	0.6 (1.4)	0.6 (1.1)	0.837
Nocturnal urine volume, mL	694.4 (268.1)	662.0 (305.1)	0.488
Twenty-four-hour urine volume, mL	1628.4 (352.8)	1516.4 (476.7)	0.157
Mean voided volume/night-time, mL	181.0 (58.2)	203.6 (80.1)	0.085
Mean voided volume/24 h, mL	128.3 (32.6)	132.2 (43.4)	0.444
Maximum voided volume, mL	287.8 (93.8)	284.2 (92.9)	0.792
Npi, %	43.6 (13.1)	44.7 (12.3)	0.614
HUS, h	2.6 (0.9)	3.4 (1.6)	0.013
First night-time voided volume, mL	208.3 (75.4)	189.0 (61.6)	0.184
OABSS			
Total score	6.9 (3.7)	5.3 (3.4)	0.006
Frequency score	0.8 (0.5)	0.8 (0.6)	0.326
Nocturia score	2.7 (0.5)	2.3 (0.9)	0.02
Urgency score	2.0 (1.8)	1.4 (1.6)	0.032
Urgency incontinence score	1.4 (1.7)	0.9 (1.3)	0.045
ICIQ-SF			
Total score	6.0 (6.0)	5.0 (4.6)	0.191
Frequency of leaks score	1.8 (1.8)	1.5 (1.5)	0.204
Amount of leaks score	1.6 (1.6)	1.4 (1.2)	0.376
Quality of life score	2.6 (2.9)	2.1 (2.5)	0.195
Ankle circumference, cm	22.5 (2.4)	21.8 (2.2)	0.006
Calf circumference, cm	33.7 (4.5)	32.5 (6.0)	0.04

Ankle and calf circumferences significantly decreased by an average of 0.7 and 1.2 cm, respectively (P = 0.006 and P = 0.040, respectively). Patient-reported satisfaction levels after four weeks of treatment were as follows: “satisfied” in five patients (16.1%), “somewhat satisfied” in 15 (48.4%), “dissatisfied” in three (9.7%), and “unanswered” by eight (25.8%).

Twenty-six patients also had nocturnal polyuria (NPi >33%). Decreases in the night-time and 24-h frequencies in the FVC in addition to prolonged HUS were observed in these patients. A slight reduction was noted in the nocturnal urine volume in patients with nocturnal polyuria (Table 3).

**Table 3:** FVC, OABSS, ICIQ-SF, and ankle/calf circumference at baseline and after four weeks in patients with nocturnal polyuria FVC: frequency volume chart; OABSS: overactive bladder symptom score; ICIQ-SF: international consultation on incontinence questionnaire-short form

N = 26; mean (SD)	Before	After	P-value
FVC			
Night-time frequency	3.0 (1.0)	2.5 (1.1)	0.014
Twenty-four-hour frequency	13.1 (3.1)	12.0 (3.5)	0.035
No. urgency episodes/24 h, N = 6	0.8 (2.0)	0.4 (1.0)	0.356
No. leaks/24 h, N = 7	0.7 (1.4)	0.7 (1.2)	0.837
Nocturnal urine volume, mL	746.5 (231.2)	686.9 (311.4)	0.223
Twenty-four-hour urine volume, mL	1585.3 (353.2)	1471.7 (401.7)	0.136
Mean voided volume/night-time, mL	191.2 (49.7)	203.8 (83.2)	0.309
Mean voided volume/24 h, mL	124.7 (33.1)	129.8 (45.5)	0.367
Maximum voided volume, mL	287.8 (95.0)	284.6 (99.9)	0.833
Npi, %	46.9 (9.1)	45.6 (12.2)	0.403
HUS, h	2.5 (0.7)	3.1 (1.5)	0.038
First night-time voided volume, mL	210.2 (78.8)	187.0 (63.9)	0.148
OABSS			
Total score	7.0 (3.8)	5.7 (3.4)	0.016
Frequency score	0.9 (0.5)	0.8 (0.6)	0.327
Nocturia score	2.7 (0.4)	2.4 (0.8)	0.058
Urgency score	1.9 (1.9)	1.5 (1.6)	0.102
Urgency incontinence score	1.4 (1.7)	1.0 (1.3)	0.116
ICIQ-SF			
Total score	6.3 (6.1)	5.3 (4.3)	0.257
Frequency of leaks score	1.9 (1.8)	1.6 (1.5)	0.539
Amount of leaks score	1.7 (1.7)	1.6 (1.2)	0.248
Quality of life score	2.7 (2.9)	2.1 (2.3)	0.264
Ankle circumference, cm	22.8 (2.3)	22.1 (2.1)	0.01
Calf circumference, cm	34.1 (4.6)	32.7 (6.3)	0.036

## Discussion

For the treatment of nocturia, behavioral therapies including lifestyle interventions, bladder training, and/or physical therapies including pelvic floor muscle training have been reported to be the treatment of choice because they are safe and cost-effective. The bladder training and physical therapies are performed with or without drug therapies for the overactive bladder or storage dysfunction. Because nocturia has been related to lifestyle disorders, various lifestyle interventions have been recommended. Among these therapies, using compression stockings has been recommended especially for nocturnal polyuria [8,9]. However, this therapy is usually performed combined with other behavioral therapies, and the effectiveness of this therapy has not been verified as a sole therapy. The present study aimed to examine the effects of wearing compression stockings as the sole therapy for nocturia, but some patients still underwent other behavioral therapies and medications.

In the present study, significant decreases were observed in the night-time and 24-h frequencies in the bladder diary. The amount of extracellular fluid that accumulated in the legs during the day correlated with the nocturnal urine volume. Although the attenuation of leg edema in the daytime was previously suggested to mitigate nocturnal polyuria, in the present study, wearing compression stockings in the daytime did not significantly decrease nocturnal urine volumes and did not alter NPi [11]. The same result was obtained when only patients with nocturnal polyuria were analyzed. A slight decrease was noted in the nocturnal urine volume, and we speculated that the lack of a significant change was due to the small number of cases or the protocol of this study not providing other lifestyle instructions. On the other hand, HUS was prolonged, which may have contributed to the decrease detected in night-time frequency. After a change to a supine position at bedtime, the movement of extracellular fluid in the legs mostly occurs early in the bedtime period, and this has been associated with the shortening of HUS [12]. In the supine position, hydrostatic pressure in the legs decreases, water in the intercellular spaces enters the circulatory system, hemodilution due to an increase in circulation occurs, antidiuretic hormone (ADH) secretion is suppressed, and nocturnal urine volume increases [13]. Therefore, the wearing of compression stockings in the daytime may have reduced the accumulation of extracellular fluid in the legs, which caused the rapid movement of extracellular fluid in the early stages of sleep, leading to an increase in HUS, thereby contributing to a decrease in night-time frequency. However, ADH was not examined in the present study. A large cohort study using wearable devices by Chapple et al. also reported that nocturia was associated with decreased sleep efficacy, prolonged periods of nocturnal arousal, and shortened HUS, which is consistent with the present results showing that a prolonged HUS reduced night-time frequency [14]. OABSS, which quantifies overactive bladder symptoms, showed better total scores, nocturia scores, urinary urgency scores, and urinary incontinence scores. The FVC suggested few actual urinary urgencies and urinary incontinence episodes, while prolonged HUS was responsible for improvements in urinary urgency and urinary incontinence scores. On the other hand, ICIQ-SF, which quantifies urinary incontinence symptoms, showed no significant improvements because only seven patients had mild incontinence in the FVC. After wearing compression stockings for four weeks, ankle and calf circumferences were significantly smaller during the daytime, suggesting that the accumulation of extracellular fluid in the legs was reduced. More than half of the patients in this study showed satisfaction or somewhat satisfaction, suggesting that this was due to prolonged HUS and decreased night-time frequency.

Vaughan et al. previously reported the effectiveness of multicomponent interventions, including the wearing of compression stockings, for nocturia. Multicomponent interventions significantly reduced night-time frequency by 0.7 episodes in the FVC [15]. In the present study, night-time frequency significantly decreased by 0.5 episodes, which we consider to be consistent with the findings of the multicomponent intervention.

Sone reported the effects of compression stockings during the day in elderly patients with leg edema. The wearing of compression stockings from waking to evening decreased night-time frequency by 1.2 episodes [10]. Our patients with nocturia included those with or without leg edema, but the ankle and calf circumferences significantly decreased by an average of 0.7 and 1.2 cm, respectively.

Since desmopressin is not recommended in some countries for the treatment of nocturia in women, the wearing of compression stockings could be a treatment option that can be done regardless of gender.

The limitations of the present study are that there were no controls because it was a pilot study. Comparative studies with a large number of patients are awaited in the future. In addition, it was difficult to evaluate the effects of compression stockings as a sole therapy, since many patients with nocturia may have been treated with various therapies including other behavioral therapies. 

In the present study, compression stockings with a pressure range between 15 and 20 mmHg were used. The effective pressure of compression stockings varies by the product and with the disease - between 25 and 35 mmHg for deep vein thrombosis and between 18 and 35 mmHg for venous ulceration [16,17]. Further studies are warranted to examine whether the pressure of compression stockings exerts different effects on nocturia.

## Conclusions

The present results suggest the effectiveness of wearing compression stockings during the day was satisfactory in most patients with nocturia, and the treatment was safely continued in patients who experienced no pain when wearing the stockings. However, because of the real-world clinical setting of this study, various confounding factors could not be excluded, and the results were not comparable to those of a placebo. Nevertheless, based on the results of this study, we believe that it is worth considering as a treatment for nocturia.
